# Predictors of inappropriate complementary feeding practice among children aged 6 to 23 months in Wonago District, South Ethiopia, 2017; case control study

**DOI:** 10.1186/s12887-019-1523-6

**Published:** 2019-05-10

**Authors:** Zerihun Berhanu, Taddese Alemu, Dirshaye Argaw

**Affiliations:** Department of Public Health, College of Medicine and Health Sciences, Dilla University, PO Box- 419, Dilla, Ethiopia

## Abstract

**Background:**

Inappropriate complementary feeding practice could result in child illness, sub-optimal growth and development. Evidence shows a huge burden of inappropriate complementary feeding practice from global to national level. But studies regarding predictors of inappropriate complementary feeding practices were scarce especially in the study area. Therefore, the aim of **t**his study was to determine predictors and community level factors associated with inappropriate complementary feeding practice among children age 6 to 23 months in Wonago district, South Ethiopia.

**Methods:**

A community based unmatched case-control study design complemented by a qualitative and dietary data was employed among children in Wonago district from April- 07 to June- 06, 2017. A total of 372 study subjects were enrolled to the study by stratified sampling technique. Data were checked, coded and entered to Epi data and exported to SPSS for analysis. Univariate, bivariable and multivariable logistic regressions analyses were applied. A p- value < 0.05 was considered as statistical significant level.

**Results:**

Paternal household decision making on feeding(AOR = 4.65, 95% CI = (1.69, 12.81)), family priority to elders during feeding(AOR = 2.35, 95% CI = (1.08, 5.14)), absence of nearby health facility(AOR = 4.15, 95% CI = (1.63, 10.55)), unplanned pregnancy (AOR = 3.45, 95% CI = (1.21, 9.85)), missing ANC(AOR = 2.71, 95% CI = (1.48, 4.96)) and missing EPI service utilization (AOR = 2.43, 95% CI = (1.34, 4.38)) were independent predictors of inappropriate complementary feeding practices. Whereas; lack of awareness, short birth spacing practice, poverty and feeding culture were community related factors. The nutrient density of complementary foods were below WHO desired density level except for energy, protein and vitamin C.

**Conclusions:**

Inappropriate complementary feeding practice was related to household feeding cultures, health service access and utilization and community related factors like awareness, poverty and low birth spacing. Complementary foods were found to have lower nutrient density than desired by WHO. Promoting community’s health service utilization and increasing awareness regarding complementary feeding were recommended.

## Background

The period from birth to two years of age is a “critical window” for the promotion of optimal growth, health and development which are directly dependent on nutrition. World Health Organization (WHO) recommends exclusive breast feeding till six months of age; then to start complementary feeding, which is the process of starting other foods and liquids along with breast milk when breast milk alone is no longer sufficient to meet the nutritional requirements of infants [1]. A complementary feeding practices commencing at 6–8 months of age while fulfilling minimum acceptable diet is considered as appropriate complementary feeding practice, but it is considered as inappropriate when it fails to fulfill either of the above indicators [2].

Globally only one in six children are receiving a minimally acceptable diet [3]. While in Eastern and South Africa it was one in ten infant and young children [3]. In Ethiopia the status of minimum meal frequency, dietary diversity and acceptable diet were 48, 4 and 4% respectively. But minimum acceptable diet reached 7% in the recent 2016 Ethiopia Demography and Health Survey (EDHS). Similarly in South Nations and Nationalities Peoples Region (SNNPR) only 2.5% of infant and young children meet minimum dietary diversity and only 2.3% of them had the minimum acceptable diet according to EDHS 2011 [4, 5].

Proper complementary feeding is important in filling energy and nutrient gaps to continue optimal growth, development and maintain health beyond six months. The amount of energy required from complementary feeding were 200, 300 & 550 kcal/day for 6–8, 9–11 & 12–23 months child respectively. Inappropriate complementary feeding practices results in total replacement of breast milk; increase risk of malnutrition, nutrient deficiencies, diarrhea and respiratory tract infections, slow growth and development; and maternal pregnancy [6–13].

Evidence is accumulated on the fact of a strong association of complementary feeding practices with sociodemographic, household, community, health service utilization and information related factors [14–23]. But most of them emphasize on the positive direction (appropriate) and measures individual indicators of complementary feeding, while inappropriate feeding culture is predominant. Additionally, as of the investigators knowledge complementary feeding was not well studied particular in Wonago district, where population density and malnutrition are high, and health service utilization is low. Therefore, this study aimed to investigate determinants and community level factors of inappropriate complementary feeding practices; and estimate energy density and nutrient adequacy of complementary feeding in the area. This made the study very important in developing strategies and policies regarding complementary feeding.

## Methods

### Study area and period

The study was conducted in Wonago district of Gedeo zone, SNNPR, Ethiopia. The district was located 13, 102 and 377 kms from the zonal, regional and national capitals, Dilla, Hawassa and Addis Ababa, respectively. The district have 17 rural and 4 urban kebeles. The latest 2016 population projection of the national statistical authority shows that the district has 156,481 total population. There are 33,294 households having 4.7 persons per household. From the total population, 91.3% dwells in the rural, while the rest 8.7% lives in urban. There are 29,780 and 3077 households in rural and urban respectively. Person per household is 4.8 and 4.4 for rural and urban respectively. According to Gedeo zone agriculture office coffee, inset, maize, teff, cabbage, sweet potato, avocado, banana, mango were among the main agricultural production of the district. The major economic activity of the area is commerce especially on coffee and product of inset. There are 6 health centers, 20 health posts and 2 private clinics in Wonago district. The study was conducted between April 07 and June 06, 2017 G.C.

### Study design

The study employed a community based unmatched case-control analytic study design. This was complemented by a qualitative data from community and dietary data from selected households.

### Sample size determination

The sample size was calculated using EPI-Info version 7 statistical software (Center for Disease Control and Prevention, Atlanta, 2005) and the largest feasible sample size was taken. The assumptions for the sample size calculation were: proportion of young children who had exposure(maternal education with secondary and above) were 17.9% among the cases and 31% among controls [24], 80% power, 95% confidence interval, 10% non-response rate and a case: control ratio of 1:1. This yielded, a total sample size of **372** (**186** cases and **186** controls). Similarly, saturation and redundancy level of information was used to limit the number of key informants and focus group discussant of the qualitative part of the study.

### Sampling technique and sampling procedures

A stratified sampling technique was employed. Initially, all kebeles in the district was stratified into urban and rural. Five rural and one urban kebeles were randomly selected. Three weeks prior to actual data collection, using 6 data collectors census was conducted on complementary feeding practices of young children aged 6–23 months using a 24 h recall dietary assessment tool in the selected kebeles. The tool was developed based on WHO core indicators used to assess complementary feeding practices of infant and young children [2]. These are introduction of solid, semi-solid or soft foods at 6–8 months of age, meal frequency and dietary diversity. The 24 h dietary intake of the children were assessed using these structured questionnaire as of the mothers report. Based on this assessment the children’s dietary intake were labeled as appropriate (control) and inappropriate (case). Appropriate when they meet all the three indicators timely introduction, minimum meal frequency and minimum dietary diversity while it was considered inappropriate when it fails to fulfill even a single indicators. From these list of identified households, a total of 186 cases and 186 controls were selected using a simple random sampling technique.

### Operational definitions

**Timely introduction of complementary feeding:** introduction of solid, semi-solid or soft foods is recommended to start at age of 6–8 months [2]**.**

**Minimum dietary diversity:** it is receiving foods from 4 or more food groups for children 6–23 months of age [2].

**Minimum meal frequency:** it is receiving solid, semi-solid, or soft foods the minimum number of times or more among children 6–23 months. The recommended number of meals per day for 6–8 months, 9–11 months & 12–23 months is 2–3 times, 3–4 times and 3–4 plus 1–2 snacks respectively [2].

**Minimum acceptable diet:** Is the combination of both minimum dietary diversity and meal frequency [2].

**Inappropriate complementary feeding practice:** complementary feeding practices that fails to fulfill either timely introduction or minimum acceptable diet.

**Cases:** Are young children (6–23 months) with inappropriate complementary feeding practices.

**Controls:** Are young children (6–23 months) with appropriate complementary feeding practices.

### Data collection procedures

Data was collected using a study format, structured, semi-structured and unstructured quantitative and qualitative data collection questionnaires. The quantitave data collection utilized a structured interviewer administer questionnaires. It do have section that address sociodemographic, household, community and health services characteristics. Under household characteristic wealth index was assessed using household asset and housing characteristics while household food insecurity was assessed using household food insecurity access scale measurement tool. While the qualitative data was collected using semi and unstructured in-depth and focus group discussion guide more focusing on community and cultural aspects. The data collection tools were prepared in English and translated to local languages, Amharic and Gede’uffa.

The energy density and nutrient adequacy of complementary foods were estimated among 15% of the sample size, using dietary assessment method. The children food intake were weighted for two days. During each meal, weight of each ingredients during preparation, final weight of the food before taken by the child and leftover weight were taken.

The quantitative data was collected using 6 data collectors who complete at least grade 10; one for each selected kebeles. While the qualitative data collection utilized a total of 7 data collectors who are health professional. Two public health professionals supervised the whole data collection process day today. All data collectors and supervises were trained for one day before preceding to data collection.

### Pre-testing

Pre-testing and standardization of the study tools was carried out on April 2017, in Chichu which is closer but outside the proposed study area. Chichu was known to share similar economic, geographic, cultural and socio-demographic characteristics with study villages. During pre-testing the questionnaire was assessed for its clarity, understandability, length, completeness, validity and reliability. A total of 37 (10% of sample size) households was selected for pre-testing.

#### Data processing and analysis

Data was checked, coded and entered to Epi data version 3.1 and exported to SPSS (Statistical Package for Social science) version 20 for analysis. Univariate analysis like mean, median and frequencies were conducted and presented using text, tables and graphs. Wealth index was computed using the principal component analysis. Then bivariable analysis was carried out to identify candidate factors associated with outcome variable for multivariable analysis. The decision was made using Odds ratio (OR) and confidence interval (CI) at 95% confidence level. Finally those predictor variables with *P* < 0.25 were entered into multivariable analysis and the final model was fitted using variables with *P* < 0.05.

Dietary data collected from sub sample two days follow up was converted to nutritional data using the Ethiopian Food Composition Table for major macro and micronutrient contents. Each nutrient amount was calculated from each ingredient of complementary foods using the conversion factor from the above table. Then total amount of each nutrients over the two days were calculated by summing individual amount of nutrient from each ingredient of each meal. The same procedure were followed to calculate the total energy of complementary foods. Nutrient densities per 100Kcal complementary food was calculated by dividing the amount of nutrients to total energy level of complementary foods(in Kcal) and multiplying by 100Kcal while energy density was calculated by dividing total energy of complementary foods in Kcal to total amount of complementary foods in grams.

Qualitative data analysis was done manually. Each audiotape interview was professionally transcribed word by word in Geddu’uffa (local language) to Amharic and then translated to English languages. Transcribed data was analyzed manually using the thematic framework analysis method.

## Results

### Characteristic of study participants

From a total of 421 children screened by the census; 213(54.9%) and 190(45.1%) children had been practicing appropriate and inappropriate complementary feeding respectively. From this 421 children, 372 children; 186 with appropriate complementary feeding practice (control group) and 186 children with inappropriate complementary feeding practice (case group) were enrolled to this analytic case-control study. The response rate of this analytical study was 100%.

#### Sociodemographic characteristics

Majority of the study subjects, 143(76.9%) from case and 123(66.1%) from the control were enrolled from rural villages. In the same way, almost all 176(94.6%) cases and 182(97.8%) controls were cared by their biological mothers. The mean age of the mothers/care takers was 28.94 ± 4.85 years and most of them 260(70%) were in the age range of 25–34 years. The mean age of the children was 16.1 ± 4.55 months while 101(54.3%) cases and 95(51.1%) controls being in the range of 18–23 months. About 98(52.7%) cases and 96(51.6%) controls were female and male respectively (Table 1).

**Table 1:** Sociodemographic characteristics of children aged 6–23 months and their family in Wonago district, South Ethiopia in 2017 G.C

Variables	Case	Control	Total
	Frequency (%)	Frequency (%)	Frequency (%)
Maternal/Care taker Age(year)			
15–24	28(15.1)	35(18.8)	63(16.9)
25–34	131(70.4)	129(69.4)	260(69.9)
35–44	27(14.5)	22(11.8)	49(13.2)
Marital status			
Married	169(90.9)	177(95.2)	346(93)
Single	3(1.6)	2(1.1)	5(1.3)
Divorced	7(3.8)	1(0.5)	8(2.2)
Widowed	7(3.8)	6(3.2)	13(3.5)
Religion			
Protestant	136(73.1)	141(75.8)	277(74.5)
Orthodox	43(23.1)	35(18.8)	78(21)
Other^a^	7(3.8)	10(5.4)	17(4.6)
Maternal/care taker Educational status			
Illiterate	63(33.9)	48(25.9)	111(29.9)
Elementary School	99(53.2)	104(56.2)	203(54.7)
Secondary School	17(9.1)	24(13.0)	41(11.1)
College & above	7(3.8)	9(4.9)	16(4.3)
Husband Educational status			
Illiterate	52(29.1)	20(11.0)	72(19.9)
Elementary School	66(36.9)	59(32.4)	125(34.6)
Secondary School	46(25.7)	83(45.6)	129(35.7)
College & above	15(8.4)	20(11.0)	35(9.7)
Maternal/care taker Occupation			
House wife	94(50.5)	79(42.7)	173(46.6)
Merchant	37(19.9)	49(26.5)	86(23.2)
Daily laborer	15(8.1)	20(10.8)	35(9.4)
Farmer	18(9.7)	22(11.9)	40(10.8)
Government employee	10(5.4)	9(4.9)	19(5.1)
Student	12(6.5)	6(3.2)	18(4.9)
Husband’s Occupation			
Farmer	69(39.2)	56(30.8)	125(34.9)
Merchant	29(16.5)	58(31.9)	87(24.3)
Daily labor	45(25.6)	25(13.7)	70(19.6)
Government employee	26(14.8)	33(18.1)	59(16.5)
Other^b^	7(3.8)	10(5.4)	17(4.8)
Child Age(month)			
6–11	30(16.1)	31(16.7)	61(16.4)
12–17	55(29.6)	60(32.3)	115(30.9)
18–23	101(54.3)	95(51.1)	196(52.7)
Family size			
1–3	13(7.0)	16(8.6)	29(7.8)
4–6	116(62.4)	121(65.1)	237(63.7)
> 6	57(30.6)	49(26.3)	106(28.5)

^a^Includes Muslims and non-religious ^b^Includes students and non-workers

#### Household related characteristics

Households with three and above under five children were 15(8.1%) and 20(10.8%) among cases and controls respectively. Mothers were the decision makers of household feeding in most of the households among both cases 150(80.6%) and controls 178(95.7%). About 81(43.5%) household had moderate food insecurity among cases while among controls 62(33.3%) household had food security. Majority of the household had access to diary (258) and flesh (350) while most of the household access vegetables from market (225) among both case and control (Table 2).

**Table 2:** Household related characteristic of children aged 6–23 months in Wonago district, South Ethiopia in 2017 G.C

Variables	Case	Control	Total
	Frequency (%)	Frequency (%)	Frequency (%)
Wealth index			
Low	61(32.8)	58(31.2)	119(32)
Medium	63(33.9)	61(32.8)	124(33.3)
High	62(33.3)	67(36)	129(34.7)
Number of children in the HH			
1–3	92(51.3)	99(53.2)	191(51.3)
4–6	83(44.6)	81(43.5)	164(44.1)
> 6	11(5.9)	6(3.2)	17(4.6)
Number of under-5 children in the HH			
One	89(47.8)	77(41.4)	166(44.6)
Two	82(44.1)	89(47.8)	171(46)
Three & above	15(8.1)	20(10.8)	35(9.4)
Availability of radio & TV in the HH			
Radio	87(46.8)	92(49.5)	179(48.1)
Television	22(11.8)	48(25.8)	70(18.8)
Household food insecurity			
Food secure	36(19.4)	62(33.3)	98(26.3)
Mild food insecure	17(9.1)	45(24.2)	62(16.7
Moderate food insecure	81(43.5)	48(25.8)	129(34.7)
Severely food insecure	52(28.0)	31(16.7)	83(22.3)
Domestic animals in the HH			
Yes	104(55.9)	91(48.9)	195(52.4)
No	82(44.1)	95(51.1)	177(47.6)
Access to animal source of food			
No	6(3.2)	3(1.6)	9(2.4)
Diary	111(59.7)	147(79.0)	258(69.4)
Egg	37(19.9)	73(39.2)	110(68)
Flesh	173(93.0)	177(95.2)	350(94.1)
Source of vegetables			
No access	18(9.7)	35(18.8)	53(14.2)
Backyard garden	43(23.1)	27(14.5)	70(18.8)
Market	110(59.1)	115(61.8)	225(60.5
Other^a^	15(8.1)	9(4.8)	24(6.5)

^a^Includes relatives and neighbors

#### Community related characteristics

There were food restriction among 15(8.1%) cases and 10(5.4%) controls. Family members other than children especially fathers were culturally preferred in getting quality food in 147(79%) cases and 124(66.7%) controls. One hundred thirty five (72.6%) cases and one hundred forty seven (79%) controls mentioned that grandmothers had no role on complementary feeding practices (Table 3).

**Table 3:** Community related characteristics of children aged 6–23 in Wonago district, South Ethiopia 2017 G.C

Variables	Case	Control	Total
	Frequency (%)	Frequency (%)	Frequency (%)
Food preference in the family			
Children less than 2 years	31(16.7)	53(28.5)	84(22.6)
Children ≥2 years	8(4.3)	9(4.8)	17(4.6)
Other family members	147(79.0)	124(66.7)	271(72.8)
Type of role grandmothers have on CF			
Insisting CF early initiation	24(47.1)	19(48.7)	43(47.8)
Insisting CF timely initiation	16(31.4)	18(46.2)	34(37.8)
Other	11(21.6)	2(5.1)	13(14.4)
Reason for CF early/late initiation			
Child could get hungry	17(53.1)	12(57.1)	29(54.7)
Mothers engaged with work	11(34.4)	4(19)	15(28.3)
Breast feeding is enough	4(12.5)	5(23.8)	9(17)

#### Health service related characteristics

Health facility was not available for 51(27.4%) cases and 10(5.4%) controls near to their village. Most of the children; 146(78.5%) cases and 178(95.7%) controls were from planned pregnancy. Only 18(9.7%) cases and 22(11.8%) controls were first birth order. Eighty seven (47%) and eighteen (10%) mothers from cases and controls respectively had no ANC visit whereas home delivery was above 40% for both cases and controls. About 18(43.5%) mothers of cases did not receive information regarding complementary feeding during MCH service utilization whereas 64(34.4%) mothers of controls received the information from ANC service (Table 4).

**Table 4:** Health service related characteristics of children aged 6–23 months and their mothers in Wonago district, South Ethiopia in 2017 G.C

Variables	Case	Control	Total
	Frequency (%)	Frequency (%)	Frequency (%)
Birth Order			
First	18(9.7)	22(11.8)	40(10.8)
2–4	115(61.8)	109(58.6)	224(60.2)
> 4	53(28.5)	55(29.6)	108(29)
MCH Service utilization for the index child			
ANC			
No visit	87(46.8)	18(9.7)	105(28.2)
< 4 visit	65(34.9)	116(62.4)	181(48.7)
4 visit	19(10.2)	17(9.1)	36(9.7)
> 4 visit	15(8.1)	35(18.8)	50(13.4)
Place of birth			
Home	99(53.2)	75(40.3)	174(46.8)
Health post	9(4.8)	16(8.6)	25(6.7)
Health center	48(25.8)	65(34.9)	113(30.4)
Hospital	30(16.1)	30(16.1)	60(16.1)
PNC	64(34.4)	114(61.3)	178(47.8)
EPI	181(97.3)	184(98.9)	365(98.1)
Growth monitoring	86(46.2)	130(69.9)	216(58.1)
Under-5 OPD	118(63.4)	132(71.0)	250(67.2)
Receive CF information from MCH services			
No	81(43.5)	14(7.5)	95(25.5)
ANC	24(12.9)	64(34.4)	88(23.7)
Delivery	23(12.4)	32(17.2)	55(14.8)
PNC	2(1.1)	3(1.6)	5(1.3)
EPI	40(21.5)	50(26.9)	90(24.2)
Under-5 OPD	6(3.2)	10(5.4)	16(4.3)
Other	10(5.4)	13(7.0)	23(6.2)

**Fig. 1:**
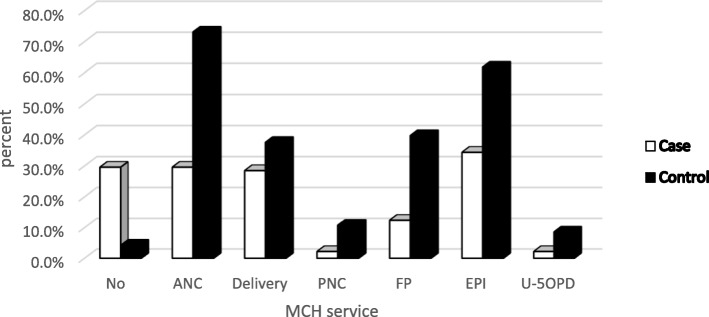
MCH service ever utilization among mothers of children aged 6–23 months in Wonago district, South Ethiopia in 2017 G.C

Whereas three mothers out of ten had no history of MCH service utilization among cases but it was only 4.3% among controls. Whereas PNC and under-5 outpatient department (OPD) were least ever utilized MCH services in both case and control groups (Fig. 1).

#### Information, knowledge and practice related characteristics

**Fig. 2:**
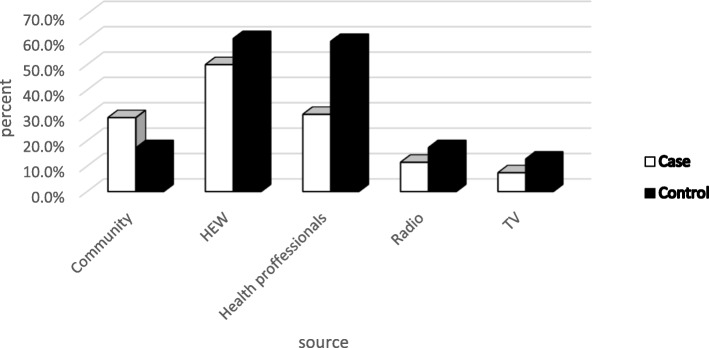
Source of information regarding complementary feeding practices in Wonago district, South Ethiopia 2017 G.C

**Fig. 3:**
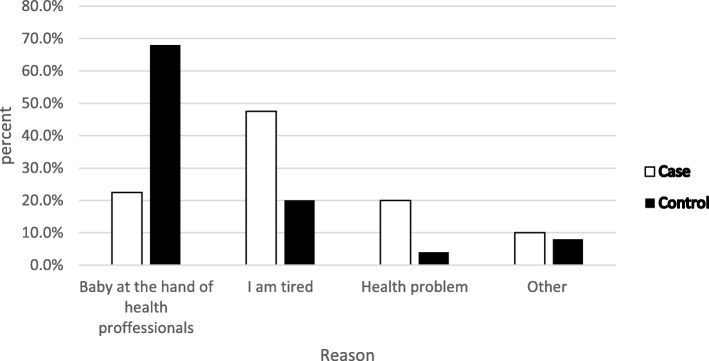
Reason of mother for late initiation of breast feeding in Wonago district, South Ethiopia 2017 G.C

A large proportion of the mothers, 105(56.5%) cases and 126(67.7%) controls had media exposure. A majority of controls (108/60.3%) than cases (74/50%) got information about complementary feeding from health workers (Fig. 2). Majority of the mothers in both groups had appropriate knowledge regarding breast feeding initiation time 264(71%), exclusive breast feeding duration 323(86.8%) and initiation time of complementary feeding 325(87.4%). Most of them practiced proper breast feeding initiation and exclusive breast feeding (Table 5). Whereas the reason for late breast feeding initiation were baby being at the hand of the health professional and being tried was mentioned by most of the mothers among control and case respectively (Fig. 3).

**Table 5:** Information, knowledge and practice regarding breast feeding and complementary feeding among mothers having children 6–23 months in Wonago district, South Ethiopia in 2017 G.C

Variables	Case	Control	Total
	Frequency (%)	Frequency (%)	Frequency (%)
Heard about CF			
No	38(20.4)	7(3.8)	45(12.1)
CF initiation time	89(47.8)	132(71.0)	221(59.4)
CF dietary diversity	62(33.3)	101(54.3)	163(43.8)
CF meal frequency	33(17.7)	54(29.0)	87(23.4)
Knowledge about BF starting time			
Within 1 h after birth	119(64.0)	145(78.0)	264(71)
After 1 h after birth	67(36.0)	41(22.0)	108(29)
Knowledge about EBF duration			
Correct	148(79.6)	175(94.1)	323(86.8)
Incorrect	38(20.4)	11(5.9)	49(13.2)
Knowledge about CF starting time			
Correct	147(79.0)	178(95.7)	325(87.4)
Incorrect	39(21.0)	8(4.3)	47(12.6)
BF initiation practice			
Within 1 h	146(78.5)	161(86.6)	307(82.5)
1-3 h	31(16.7)	24(12.9)	55(14.8)
> 3 h	9(4.8)	1(0.5)	10(2.7)
EBF practice			
Yes	121(65.1)	108(58.1)	229(61.6)
No	65(34.9)	78(41.9)	143(38.4)

### Predictors of inappropriate complementary feeding practice

Finally household decision maker regarding feeding, cultural preference to get food, nearby health facility, type of pregnancy, ANC and EPI service utilization were found to be independent predictors of inappropriate complementary feeding.

Children from household with paternal decision making regarding feeding were 4.7 times exposed to inappropriate complementary feeding compared to those from household with maternal decision making (AOR = 4.65, 95% CI = (1.69, 12.81)). Children in the household with preference to family members other than children for food were 2 times more likelihood of inappropriate complementary feeding practice than those in the house with preference for children less than 2 years (AOR = 2.35, 95% CI = (1.08, 5.14)).

Children from area without nearby health facility had 4 times more likelihood of inappropriate complementary feeding (AOR = 4.15, 95% CI = (1.63, 10.55)). Unplanned children had 3 times more likelihood of inappropriate complementary feeding compared to planned children (AOR = 3.45, 95% CI = (1.21, 9.85)). Those children from mothers without any history of ANC service utilization had 2.7 times higher probability of inappropriate complementary feeding as compared to children from mothers with ANC service utilization (AOR = 2.71, 95% CI = (1.48, 4.96)). Similarly children from those mother’s without history EPI service utilization had 2.4 times risk of having inappropriate complementary feeding practices compared to those from mother’s with EPI service utilization (AOR = 2.43, 95% CI = (1.34, 4.38)) (Table 6).

**Table 6:** Independent predictors of inappropriate complementary feeding practices among children 6–23 months of age in Wonago district, South Ethiopia 2017 G.C

Variables	Case n(%)	Control n(%)	COR(95%CI)	AOR(95%CI)
HH decision maker regarding feeding				
Mother	150(80.6)	178(95.7)	1	1
Father	36(19.4)	8(4.3)	5.34(2.41, 11.84)**	4.65(1.69, 12.81)**
Food preference in the family				
Children less than 2 years	31(16.7)	53(28.5)	1	1
Children ≥2 years	8(4.3)	9(4.8)	1.52(0.53, 4.35)	0.61(0.16, 2.38)
Other family members	147(79.0)	124(66.7)	2.03(1.23, 3.35)**	2.35(1.08, 5.14)**
Availability of nearby health facility				
Yes	135(72.6)	176(94.6)	1	1
No	51(27.4)	10(5.4)	6.65(3.26, 13.58)**	4.15(1.63, 10.55)**
Type of pregnancy resulting that child				
Planned	146(78.5)	178(95.7)	1	1
Unplanned	40(21.5)	8(4.3)	6.1(2.77, 13.43)**	3.45(1.21, 9.85)**
ANC service ever utilized				
Yes	55(29.6)	136(73.1)	1	1
No	137(70.4)	50(26.9)	6.48(4.12, 10.18)**	2.71(1.48, 4.96)**
EPI service ever utilized				
Yes	64(34.4)	115(61.8)	1	1
No	122(65.6)	71(38.2)	3.09(2.02, 4.71)**	2.43(1.34, 4.38)**

1). 1:- Reference group **:- *p*-value< 0.05 *:- *p*-value< 0.25 ^**+**^**:-** Includes fathers, aunt, grandmothers and servant

2). Independent predictors controlled for:- child caretakers, maternal age, husband education status, household food insecurity, access to diary products, access to egg, grandmothers role on CF, family planning ever utilized, received information about CF from MCH, heard about CF

### Estimated energy density and nutrient density of complementary foods

After sub-sample assessment of complementary foods; mean of estimated energy density per day was 0.865 ± 0.15 Kcal/g for 6–8 months, 0.974 ± 0.19 Kcal/g for 9–11 months and 1.081 ± 0.2 Kcal/g for 12–23 months children. While mean protein density per 100Kcal energy of complementary food per day were 1.224 ± 0.46 g for 6–8 months, 1.128 ± 0.31 g for 9–11 months and1.267 ± 0.34 g for 12–23 months children. Whereas mean calcium density per 100Kcal energy of complementary food taken by children 6–8, 9–11 and 12–23 months per day were 13.957 ± 3.47,12.023 ± 2.73 and 10.459 ± 3.07 mg respectively. Except energy, protein, vitamin C densities other nutrient densities were below WHO desired density level [25, 26] (Table 7).

**Table 7:** Estimated nutrient density per 100 kcal energy of complementary food per day for sub-sampled of 6–23 months children included in the study in Wonago district, South Ethiopia in 2017 G.C

Nutrient	6–8 months			9–11 months			12–23 months		
	Desired density	Mean	Median	Desired density	Mean	Median	Desired density	Mean	Median
Protein(g)	1	1.224		1	1.128		0.9	1.267	
Calcium(mg)	105	13.957		74	12.023		63	10.459	
Inadequate intake (%)	86.7			83.8			83.4		
Phosphorous(mg)	114	30.15		70	30.085		26	31.841	
Inadequate intake (%)	58.2			57					
Iron(mg)	4.5		1.041	3		0.964	1		0.914
Inadequate intake (%)	76.9			67.9			8.6		
Vitamin A(μg)	31	0.344		30	0.234		23	0.272	
Inadequate intake (%)	98.9			99.2			98.8		
Thiamine(mg)	0.08	0.026		0.06		0.03	0.07	0.031	
Inadequate intake (%)	67.5			50			55.7		
Riboflavin(mg)	0.08	0.055		0.06		0.038	0.06	0.04	
Inadequate intake (%)	31.3			36.7			33.3		
Niacin (mg)	1.5	0.226		1	0.239		0.9	0.271	
Inadequate intake (%)	84.9			76.1			69.9		
Vitamin C(mg)	1.5		1.647	1.7	1.563		1.5		1.590
Inadequate intake (%)				8.1					

### Qualitative study findings

#### Study participants

A total of four focus group discussions (FGD) and six in-depth interviews were conducted with purposefully selected mothers and key informants respectively. FGDs were held with thirty five mothers (7, 9, 11 & 8 mothers in each groups) having child beyond six months in the study community. In terms of their sociodemographic characteristics, three quarter of the mothers were from rural area and 68% of them had less than primary educational status. Almost all of the mothers were married and half of them were house wives. They had an average age of 29 years (range, 22 to 50 years) and an average of four children each. The in-depth interview was carried out with health professionals acting as district health office head, health center head, under five outpatient case team coordinator, MCH case team coordinator and health extension worker. Most of them were clinical nurse and diploma holders.

#### Awareness about complementary feeding

Study participants had divergent views about appropriate as well as inappropriate complementary feeding practices. Majority of key informants and the FGD discussants had good understanding on complementary feeding while others describing it poorly and often wrongly. Accordingly, four key informants and about 70% of FGD participants involved in the study described complementary feeding as a balanced food given to children with breast milk from 6 to 23 months from local resource. On the other hand, two of key informants also described it as a food given to malnourished children and a food that is given to all under five children. One of the key informants also believe that the type and amount of complementary feeding depends on growth and development of the child.

Reporting on inappropriate complementary feeding practices, most of the participants believe that complementary food could be considered inappropriate if it lacks adequacy in amount, regardless of its variety, frequency, consistency, and timely initiation.

Most participants from both FGD and in-depth interview reported that inappropriate complementary feeding has several consequences and effects on the health and development of the children that potentially leads to causes massive burden on the national economy. They emphasized its effect on the child’s health and wellbeing by affecting the immunity and disease resistance capacities.

### Burden and extent of inappropriate complementary feeding practices in the area

As most of the study participant mentioned the burden and extent of inappropriate complementary feeding practices were sever in the area. Almost all of the health professionals agreed that complementary feeding practices in the district was inappropriate being worst in the rural area. As they mentioned the community put the child on family food like qocho (local food prepared from inset) and even they don’t care whether the child eat or not.

Supporting this most of the FGD participants agreed as they didn’t provide any thing especial other than family foods. Most of the time even they provide dry type of food like qocho and qoqor (food made from bread) with tea. Some of the mothers said that they didn’t give any thing for the child except breast until above 12 month and while others start complementary feeding before six months even before three months for seek of work. Contrary to this some of FGD participants prepare different food for the child other than family food as affordable and they didn’t provide qocho and flesh till 12 months. They provide porridge prepared from maize, cow milk, egg with potato, carrot with potato, gruel and fruit like avocado, mango and banana for the child. They serve their child above two times a day as he/she needs.

### Reasons for inappropriate complementary feeding practices

The study participants from both in-depth interview and FGD list out different reasons for inappropriate feeding practices in the area. For instance most of the participants agreed that inappropriate complementary feeding result from lack of awareness, short birth spacing and poverty. But some of key informants and participants in FGD complain as it result from inappropriate usage of resource and lack of saving culture. For instance there is ample amount of different fruit source but not utilized in the home, as mentioned by most of the participants fruits were sold in the market and in return they bought inset for qocho preparation. Adding to this the area was not lucky in crop production and this was one of the reason as the participant believed. Lack of emphasis for the child in the community was one of the reason mentioned by the participants and this was demonstrated by that most of the child in the area were cared by others due to most mothers in the area were merchant.

### Culture

In both FGD and in depth interview it were believed that culture pays a major role in child feeding practices. Feeding culture of the community is not in favor of appropriate child feeding practices, for instance the staple food of the area was qocho made from inset which is not comfortable for child especially at the beginning of complementary feeding and even they depraved locally available fruits and eggs by taking to the market for the sake of earning money for inset buying. Most of the participant also revealed that as there was priority for elders especially husbands during feeding and it was mentioned that leftover was served for the child. Contrary to this some of the participant also mentioned that as there was a culture of feeding together which again also made difficult for the child to get appropriate complementary feeding putting them on ordinary family food and even they can’t compete to get adequate food. Most of the key informants also mentioned that there were a cultural medicine which made child depraved of appropriate care and treatment during sickness. So the health professionals believe this had influence on complementary feeding by suppressing child’s appetite. In contrast to the above some of the key informants believe that the culture of the community had no contribution for inappropriate complementary feeding in the area.

## Discussion

Inappropriate complementary feeding practice among children increase 4.7 times when household decision regarding feeding made by father which is in line with a study from northwest Ethiopia [15]. This can be due to paternal knowledge regarding feeding was limited especially on child feeding and most of the food preparation also carried out by mothers. This was evident that small portion of the study participants were cared by family members other than mothers which includes fathers, grandmothers and servants.

The culture of preference for family members other than children during feeding was responsible for increasing the odds of inappropriate complementary feeding practice among children by 2.4. This is supported by the finding from qualitative study in which feeding culture of the community was mentioned as a responsible factor for inappropriate complementary feeding practices. Among these cultures priority during feeding was given for elders and leftover was served for children in the study area as revealed from in-depth interview and focus group discussion. On the other hand there was a culture of serving all family members including children together during feeding putting them on ordinary family food. In addition to this the qualitative result showed that children were a community members whom lacks due attention/emphasis by the community. But as of the investigator knowledge this family preference during feeding was not mentioned in other studies.

The odds of inappropriate complementary feeding practices rise by 4.2 when nearby health facility was not available. This may be related with as the facility is nearest to the community, the culture of service utilization increases which intern improve the awareness including about complementary feeding. This was not assessed by other studies as of the investigators knowledge.

In this study unplanned pregnancy increase the risk of inappropriate complementary feeding practices 3.5 times compared to planned one. During planning to have a child, the family will prepare themselves in different ways for instance psychological and economical preparation. But in the case unplanned pregnancy this preparation will be missed. Consequently the child may be depraved of proper care including feeding. Short birth spacing was one of the factor mention in the qualitative result which was interrelated with unplanned pregnancy. During short birth spacing there was shortage of resource which may result in feeding problem of the children. In contrast study from Mekelle town revealed that whether the child was from planned or unplanned pregnancy it did not have statistical significance on timely initiation of complementary feeding [22]. This difference may be due to this two studies were different in design and study setting.

As of this study mothers without exposure to ANC and EPI service were found engage their children with inappropriate complementary feeding practices. This is due to improved awareness gained during these service utilization. Similarly studies from South Asia countries revealed inadequate antenatal care as a predictor of inappropriate complementary feeding practices [27, 28]. Moreover, studies from Ethiopia [18, 20, 22] and Nepal [21] revealed that ANC service utilization had improved complementary feeding practices. In contrast other cross-sectional studies in Ethiopia [16, 19, 23, 29] sated that ANC service utilization had no statistical significance on commencement of complementary feeding but they differ from this study in design and focusing point. Whereas as far as the knowledge of the investigator there was no evidence regarding the influence of EPI service utilization on complementary feeding practices.

Lack of awareness was one of the factor for inappropriate complementary feeding according to the qualitative result. This is in line with a quasi-experimental study from Dilla showed improvement in complementary feeding practice after intervening with nutritional education [30]. Similarly studies from Dangila [15], Jima [23] and Bishoftu [19] revealed that having media exposure and information regarding breast feeding improved complementary feeding practices.

Poverty was among the factor mentioned for inappropriate complementary feeding during focus group discussion whereas wealth index lack statistical significance in the quantitative part. This is in line with study in south Wollo where middle-income and rich households had improved complementary feeding practice in respect to dietary diversity [14]. Moreover, food availability and affordability was one of the factor determining complementary feeding practices in Zambia [31]. This is because economic status play role in fulfilling household resource including ingredients for complementary food preparation. So that poverty can limit accessing those resource and result inappropriate complementary feeding practice. Inline to quantitative result income was not statistically significant with appropriate complementary feeding practice in studies at Sidama zone and Abyi-Adi town [17, 32].

According to the qualitative result improper resource utilization was related to inappropriate complementary feeding practices. For instance the area is rich in different fruits but as mentioned in the focus group discussion and in-depth interview it was sold in the market and not served for the children. As it was recommended by WHO serving fruits can improve complementary feeding practices [2].

Only energy, protein and vitamin C densities were above WHO desired density level [25, 26]. This is because maize, meat, qocho and kidney beans are among commonly utilized foods in the area and they are good in energy and protein according to Ethiopian food composition table. Whereas green pepper may be responsible for vitamin C as it is commonly utilized in the area [33]. Similarly complementary food protein density was above desired level in other studies from Ethiopia [34, 35].This finding is in line with finding from South Africa, Guatemalan and Peru in respect to protein and vitamin C densities [36–39]. In contrast vitamin C density was below desired level in Wollo and Sidama of Ethiopia [34, 35]. This may be due to difference in the way of dietary analysis, since this two studies followed biochemical analysis.

On other hand thiamine and riboflavin densities of complementary food was below the desired density of WHO. Whereas the density for niacin and iron was lower than desired density level [25, 26]. While phosphorus, calcium and vitamin A densities were far below desired density level. These may be due to lower amount of complementary food served per day. Similarly thiamine and riboflavin densities of complementary foods in Sidama zone Ethiopia were below desired level [34]. Niacin and vitamin A densities of complementary food in Ethiopian and Guatemalan were similarly below the desired density level [34, 35, 37]. Furthermore, studies from Ethiopia, South Africa, Guatemalan and Peru also revealed that iron and calcium densities were below WHO recommendation [34–39]. Densities for thiamine, riboflavin, niacin and vitamin A were above the desired recommendation level in Guatemalan [36]. This difference may be explained by the difference in feeding culture of two community.

As a limitation most of the variables were measured based on subjective report of the participants which may introduced recall bias. Estimating energy and nutrient density of complementary foods using sub-sample without biochemical analysis was another limitation of the study.

## Conclusions

According to this study inappropriate complementary feeding practices was related to household feeding cultures, health service access and utilization and community related factors like awareness, poverty and low birth spacing. Complementary foods were found to have lower nutrient density than desired by WHO. Promoting community’s health service utilization and increasing awareness regarding complementary feeding are recommended.

## Abbreviations

ENA: Emergency nutrition assessment
EPI: Expanded program of immunization
HMIS: Health management information system
MCH: Maternal and child health
OPD: Out patient department
OR: Odds ratio
RNI: Recommended nutrient intake
